# Oil-Based Phase Change Emulsions Endowed with High Thermal Conductivity and Responsive Rheological Behavior

**DOI:** 10.3390/ma19071330

**Published:** 2026-03-27

**Authors:** Yihua Qian, Qing Wang, Yaohong Zhao, Zhi Li

**Affiliations:** Guangdong Key Laboratory of and Electric Power Equipment Reliability, Electric Power Research Institute of Guangdong Power Grid Co., Ltd., Guangzhou 510080, China

**Keywords:** phase change emulsion, oil-soluble, thermal conductivity enhancer, non-Newtonian fluid

## Abstract

To overcome the low thermal conductivity and flow channel clogging inherent in traditional phase change materials (PCMs) for immersion cooling, this study develops a novel oil-based phase change emulsion (PCE) integrating high thermal transport with adaptive rheological behavior. A liquid thermal conductivity enhancer was synthesized by modifying epoxidized soybean oil with LiTFSI and blending it with a synthetic ester to form a dielectric base fluid. A mid-to-low-temperature PCM (Span65) was then incorporated via surfactant-free ultrasonic emulsification. The resulting PCE exhibits a tunable phase-change window (25~40 °C) driven by interfacial confinement effects and a multiscale lamellar network. It achieves significantly enhanced thermal conductivity (15% increase over base oil) while maintaining excellent electrical insulation (<10^−9^ S/cm). Rheologically, the emulsion transitions from shear-thinning in the solid state to near-Newtonian in the liquid state, optimizing both suspension stability and pumping efficiency. This work establishes a strategy for designing high-performance, safe, and energy-efficient dielectric coolants, offering a robust solution for next-generation electronic and battery thermal management systems.

## 1. Introduction

As information technology increasingly converges on high-performance computing, artificial intelligence inference, and large-scale cloud services, the power consumption of individual machines has surpassed the kilowatt threshold [1,2]. Traditional air-cooling or water-cooling technologies are progressively inadequate for meeting the stringent thermal dissipation demands of such systems [3,4]. Phase Change Material (PCM) cooling technology exploits the characteristic of PCMs to absorb substantial latent heat during phase transitions while maintaining a nearly constant temperature, thereby achieving effective thermal management [5,6]. Compared with air cooling, PCM cooling possesses a higher volumetric heat capacity; in contrast to heat pipes, it features a simplified system architecture and more uniform temperature distribution; and relative to forced liquid cooling, it exhibits significantly reduced energy consumption, thus attracting extensive research interest [7,8].

Nevertheless, the practical application of PCMs encounters several challenges. Their inherently low thermal conductivity tends to induce significant temperature gradients, leading to localized heat accumulation and exacerbating system overheating [9]. Furthermore, within cooling circulation systems, the solidified PCM post-phase transition may aggregate in low-temperature regions, potentially causing flow obstruction and ultimately resulting in cooling system failure. To address these issues, incorporating PCM into a coolant medium in the form of an emulsion to create Phase Change Emulsions (PCEs) has emerged as a critical research direction within the realm of immersion cooling [5,10,11].

Direct liquid cooling, commonly referred to as immersion cooling, offers a thermally efficient solution characterized by a simplified system architecture, low thermal resistance, and high heat removal capacity [12]. This approach employs a heat transfer fluid (HTF) to extract thermal energy directly from battery cells, thereby enabling faster and more compact thermal regulation compared to conventional air-based battery thermal management systems (BTMS) [13]. An ideal HTF for such applications must exhibit superior thermophysical properties—including high thermal conductivity, low dynamic viscosity, low density, and favorable specific heat capacity—while remaining chemically inert toward components to preclude adverse reactions [14]. Historically, water, mineral oils, and silicone oils have been the predominant choices as coolants in direct-contact configurations. Although water possesses excellent thermal properties, its electrical conductivity and reactivity necessitate stringent sealing measures. In contrast, mineral oil– and silicone oil–based systems, despite their comparatively lower thermal conductivity, offer inherent electrical insulation, chemical stability, and non-corrosive behavior [15]. Consequently, when the coolant is in direct contact with battery modules, these dielectric oils enable simpler, more compact system designs that significantly reduce both manufacturing complexity and long-term maintenance costs.

Currently, immersion cooling dielectrics primarily encompass mineral oils [16], silicone oils [17], vegetable oils [18], nanofluids [19,20,21,22], and synthetic ester-based oils [23,24,25]. Among these, synthetic esters have garnered considerable interest owing to their high chemical purity, tunable molecular architecture via controlled industrial synthesis, and favorable biodegradability profile [26,27].

Despite these advantages, conventional synthetic ester-based coolants remain constrained by their intrinsically low thermal conductivity, which limits overall heat transfer efficiency and necessitates performance enhancement. In recent years, significant progress has been made in augmenting coolant thermal conductivity through the incorporation of thermal conductivity enhancers (TCEs). For instance, Chen et al. [28]. comprehensively reviewed that the addition of carbon-based nanomaterials—such as graphene and carbon nanotubes—to base fluids can elevate thermal conductivity by 30%–120% through the formation of percolating thermal pathways, with minimal impact on viscosity. Similarly, dispersing metallic (e.g., Cu, Ag) or metal oxide (e.g., Al_2_O_3_, TiO_2_) nanoparticles at volume fractions ranging from 0.1% to 6% into aqueous or ethylene glycol-based carriers has yielded thermal conductivity improvements of 10–60% [28]. Direct incorporation of solid salts or nanoparticles frequently results in agglomeration, sedimentation, and insulation degradation [29]. Thus, the development of a liquid, single-phase TCE is essential to ensure long-term colloidal stability and dielectric safety.

In this study, we propose a novel liquid TCE. Unlike conventional modifiers, ESO possesses a molecular structure analogous to that of the base oil, ensuring exceptional compatibility. This intrinsic compatibility guarantees a homogeneous and stable liquid phase even at high concentrations, effectively preventing precipitation. Subsequently, by incorporating this modified TCE into a synthetic ester-based base fluid, we achieved an optimal balance between viscosity and thermal conductivity. Building upon this thermally enhanced matrix, low-temperature PCM (Span 65) was encapsulated via high-energy emulsification to engineer an intelligent cooling fluid exhibiting a gradient. This design strategy aligns perfectly with the principle of “enhancing functional properties without compromising core performance.” To realize this objective, we systematically investigated the influence of processing parameters on emulsion characteristics. Through a comprehensive suite of characterizations—including thermophysical property analysis, microstructural morphology assessment, rheological profiling, and long-term stability evaluation—we established robust structure–property–performance relationships. These findings provide new insights and a theoretical foundation for the development of next-generation immersion cooling systems.

## 2. Experimental

### 2.1. Preparation of Low-Temperature Nano-Phase Change Emulsions

#### 2.1.1. Materials

The synthetic ester oil (SE), employed as the base coolant fluid, was procured from Lubemater Co., Ltd., Qingdao, China. Lithium bis(trifluoromethanesulfonyl)imide (LiTFSI) was sourced from Morni Chemical Co., Ltd., Shanghai, China. Sorbitan tristearate, utilized as PCM, and epoxidized soybean oil, serving as the thermal conductivity enhancer (TCE), were both obtained from Macklin Biochemical Co., Ltd., Shanghai, China and Aladdin Reagent Co., Ltd., Shanghai, China, respectively. All chemicals were used as received without further purification.

#### 2.1.2. Preparation of Modified TCE (EF)

A total of 96.0 g of epoxidized soybean oil was heated to 60 °C, followed by the addition of 4.0 g of LiTFSI. The reaction mixture was continuously stirred in an oil bath maintained at 60 °C for 2 h until a clear, transparent solution was obtained. Subsequently, the solution was subjected to vacuum drying at 80 °C for 12 h, yielding a thermal conductivity enhancer with a LiTFSI loading of 4 wt%. Using an identical protocol, a series of TCE modified with LiTFSI (EF) samples with LiTFSI concentrations of 2, 1, 0.2, and 0.02 wt% were synthesized. These EF formulations were then incorporated into a synthetic ester base oil at weight fractions of 20, 40, 50, 60, and 80 wt%, respectively. Each blend was thoroughly homogenized under mechanical stirring to produce a series of thermally enhanced cooling fluids.

### 2.2. Preparation of PCEs

The phase change emulsion (PCE) was prepared via ultrasonic emulsification, with Span 65 (PCM) incorporated at mass loadings of 4, 6, 8, and 10 wt%. The procedure was carried out as follows: the Span 65 was first heated to its fully molten state and subsequently introduced into the base fluid (SE, EF, or SE–EF blend). The emulsification was then subjected to mechanical stirring at 105 °C and 400 rpm for 20 min to establish a preliminary homogeneous dispersion.

Thereafter, the vessel was immediately immersed in an ice bath to promote rapid interfacial solidification. Simultaneously, a probe ultrasonic cell disruptor (LS-1200B, Yuanshengte Intelligent Technology Co., Ltd., Wuxi, China) was inserted into the fluid. Sonication was performed at 50% amplitude (400 W output) in a pulsed mode (30 s on/30 s off) for 20 min. The system operated at a constant frequency of 20 kHz with a 10 mm diameter probe. This controlled protocol facilitated the formation of stable, nanostructured PCEs with uniform droplet morphology.

A pivotal innovation in this work is the dual function of Span 65 as both the primary PCM and an intrinsic surfactant, circumventing stability challenges associated with conventional additives. Distinct from traditional systems relying on separate additives, Span 65 in our formulation orchestrates two critical mechanisms:

Lipophilic Emulsification: With a low Hydrophilic-Lipophilic Balance (HLB ≈ 2.1), Span 65 is ideally suited for stabilizing solid-in-oil systems. During high-shear homogenization, Span 65 molecules rapidly adsorb at the interface between molten droplets and the continuous EF base fluid, forming a robust protective layer.

Active Participation in Phase Transition: As a fatty acid ester, Span 65 undergoes crystallization during cooling. This in situ crystallization fosters a semi-rigid, surfactant-rich interfacial shell, augmenting latent heat capacity and reinforcing mechanical integrity against shear-induced deformation.

### 2.3. Characterization

Kinematic viscosity was measured in accordance with ASTM D445 [30] using an automated capillary viscometer (S-FLOW1200, Hangzhou Yungyi Technology Co., Ltd., China) at 40 °C. Electrical conductivity was determined at 25 °C per ASTM D1125 [31] using a digital conductivity meter (DDSJ-308A, INESA Scientific Instrument Co., Ltd., China) with a platinum black electrode. Thermal conductivity was quantitatively determined using the transient hot wire (THW) method (ASTM D7896–23 [32]) with a thermal conductivity meter (TC3000E, XIATECH Electronic Technology Co., Ltd., China) at 25 °C. Samples were degassed prior to measurement.

Morphological analysis was conducted using a field-emission scanning electron microscope (FE-SEM, Zeiss Sigma 560, Carl Zeiss Microscopy GmbH, Germany) at 3–5 kV. Optical micrographs were obtained using an polarized light microscope (BX41, Olympus, Japan). Thermal behavior was monitored using a differential scanning calorimeter (DSC 214, NETZSCH, Germany). The protocol involved heating from 0 °C to 80 °C and cooling back to 0 °C at 5 °C/min. Oxidation stability was evaluated via Pressure DSC (DSC 204 HP, NETZSCH, Germany) under 35 bar nitrogen pressure.

The rheological properties of the greases were characterized using an rotational rheometer (MCR 302, Anton Paar GmbH, Austria) equipped with a PP25/TG parallel plate (25 mm diameter) at a fixed gap of 1 mm. At 20, 30 and 40 °C, the apparent viscosity was measured as the shear rate gradually increased from 0.01 to 1000 s^−1^ to evaluate the shear-thinning behavior of the samples. Samples were first melted at 50 °C (5 min), then quenched to 0 °C (5 min) to ensure intimate contact with the geometry, followed by equilibration at the target test temperature (≥10 min) before ramping.

## 3. Results and Discussion

### 3.1. Modification of TCE

**Figure 1:**
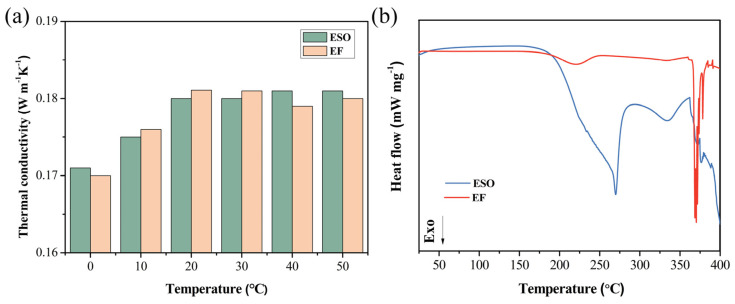
Temperature-dependent thermal conductivity (**a**) and thermal stability (**b**) of pristine and modified ESO (EF).

Figure 1 illustrates the temperature-dependent thermal conductivity and thermal stability of pristine and modified ESO (EF). As shown in Figure 1a, thermal conductivity exhibits a marginal increase with temperature, which becomes negligible above 20 °C. Crucially, the negligible deviation between pristine and modified ESO confirms that the modifier preserves the intrinsic heat transfer capabilities of the base fluid. Thermal stability analysis (Figure 1b) reveals that unmodified ESO exhibits a pronounced exothermic peak above 200 °C, indicative of oxidative degradation. In stark contrast, EF (containing 0.02 wt% LiTFSI) exhibits an enhanced oxidative onset temperature exceeding 350 °C, demonstrating superior thermochemical stability essential for high-power-density applications.

**Figure 2:**
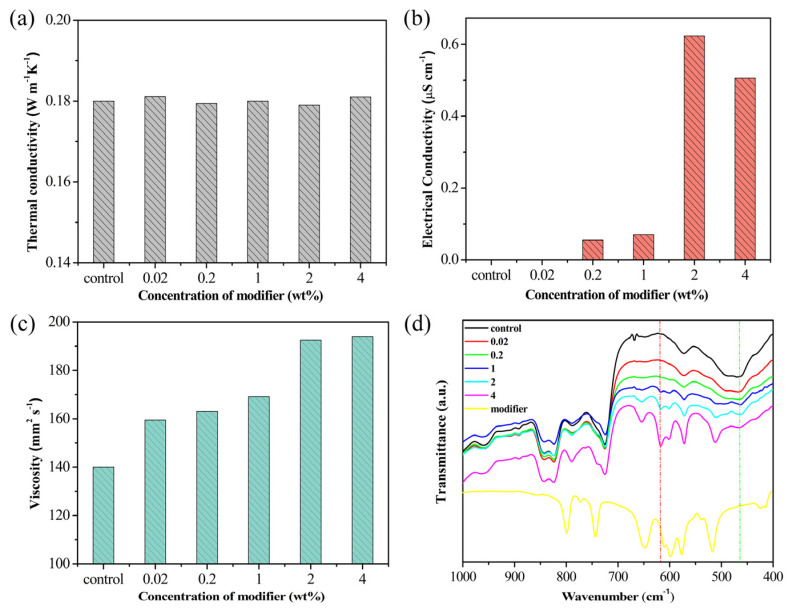
Effect of modifier (LiTFSI) concentration on the thermal conductivity (**a**), electrical conductivity (**b**), viscosity (**c**), and FTIR spectra (**d**) of EF (containing 0.02 wt% LiTFSI).

With increasing modifier concentration, EF exhibited a marked rise in electrical conductivity and viscosity, without significant reduction in thermal conductivity (Figure 2a). To balance functional enhancement with dielectric safety, the doping concentration must be strictly controlled. At 0.02 wt% LiTFSI, EF achieved an electrical conductivity below 1 × 10^−9^ S/cm—well beneath the insulation threshold for electronic cooling (<1 × 10^−5^ S/cm) [33,34,35]. FTIR spectroscopy (Figure 2d) provides direct evidence of successful chemical modification: a new characteristic peak emerges at 617 cm^−1^ in EF, attributed to the coordination interaction between Li^+^ ions and epoxy groups. Control experiments with physical mixtures confirmed that this spectral evolution arises from chemical transformation rather than physical dissolution (Figure S1).

**Figure 3:**
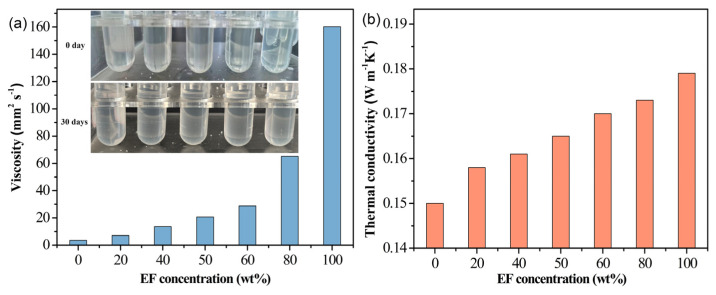
The solubility (inset in (**a**)), viscosity, and thermal conductivity (**b**) of EF in synthetic ester oil as a function of their compositional ratio.

Figure 3 demonstrates that EF exhibits complete molecular-level miscibility with the base oil across a wide concentration range, maintaining optical clarity even after 30 days of storage. This confirms exceptional compatibility, stemming from structural similarity and polarity matching. Quantitative analysis reveals critical trade-offs: increasing EF content from 50 wt% to 60 wt% raises kinematic viscosity by 40% (20.64 to 28.78 mm^2^/s) while improving thermal conductivity by only 2.5%. Further elevation to 80 wt% causes a dramatic viscosity surge (126% increase) for a mere 1.7% thermal gain. Consequently, the 60 wt% EF formulation (designated EF6) was selected as the base fluid, offering a 15% higher thermal conductivity than neat synthetic ester with acceptable rheological performance.

### 3.2. Thermodynamic Behavior Analysis of the PCE

**Figure 4:**
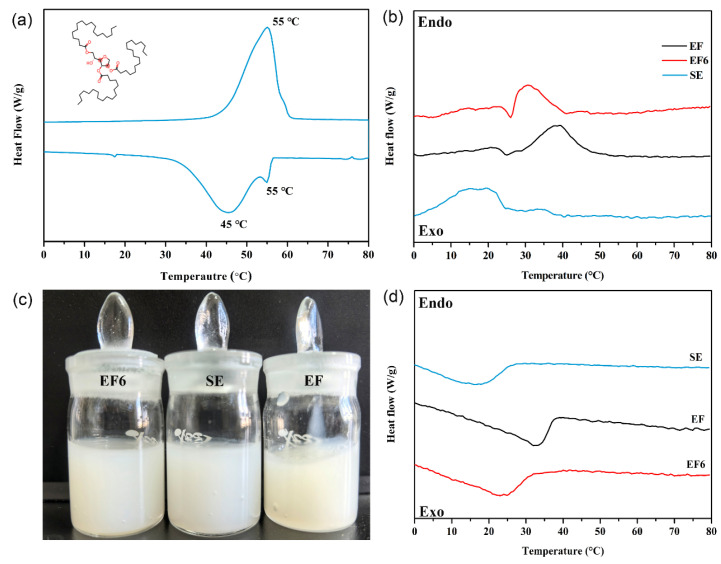
DSC thermograms of Span65 and its physical state and thermal behavior when dispersed in different base fluids—SE, EF, and EF6: (**a**) DSC thermogram of pure Span65; (**b**) DSC endothermic curves of Span65 dispersed in SE, EF, and EF6; (**c**) Photographs of Span65 dispersed in SE, EF, and EF6; (**d**) DSC exothermic curves of Span65 dispersed in SE, EF, and EF6. (The upward peaks correspond to endothermic events during heating, while the downward peaks represent exothermic events during cooling.).

Accurate characterization of Span 65 thermal behavior is pivotal for elucidating correlations among flow properties and phase transition ranges. DSC analysis (Figure 4, Table 1) reveals significant modulation effects of different matrices.

Conversely, the offset temperature is determined by the intersection of the baseline with the tangent to the trailing edge of the peak, signifying the critical endpoint of the transformation. The peak temperature, corresponding to the global extremum of the heat flow curve—minimum for endothermic (melting) and maximum for exothermic (solidification) processes—represents the temperature at which the phase change rate is maximal. For thermograms exhibiting multiple peaks, the peak temperature of the dominant phase transition event is identified by the most pronounced global extremum. The degree of supercooling is quantified as the difference between the melting peak temperature and the solidification peak temperature [36], representing the extent to which the system deviates from thermodynamic equilibrium during cooling. This parameter directly influences temperature control precision and cycling stability in practical thermal management applications.

The DSC heat flow curves of all samples and their corresponding phase change fluids are presented in Figure 4. Following standard instrument convention, endothermic processes (e.g., melting) are represented by negative heat flow signals, whereas exothermic processes (e.g., solidification) appear as positive signals. This comprehensive thermal history elimination and cyclic testing strategy enables rigorous evaluation of PCM phase transition kinetics, latent heat capacity, and cycling reversibility, thereby providing essential foundational data for subsequent rheological–thermal coupled performance analyses.

Pure Span65 exhibited excellent thermal stability and reversibility. Melting occurred between 40 and 60 °C, aligning with moderate-temperature applications like electronic cooling and waste heat recovery. Solidification peaks ranged from 56.4 to 33.3 °C, indicating a ~10 °C supercooling degree attributed to high nucleation barriers. Notably, a shoulder peak near 55 °C during cooling suggests early local crystallization triggered by homogeneous nucleation or impurities, which helps mitigate deep supercooling and enhances heat release controllability.

Using a “high-energy emulsification–ultrasonic temperature-controlled crystallization” process, Span65 was dispersed into three matrices—Synthetic Ester (SE), Electrolyte Fluid (EF), and an optimized composite (EF6)—to evaluate matrix-dependent thermal modulation:

In the SE matrix, strong interfacial interactions depressed the phase change temperature significantly (melting < 25 °C; solidification ~20 °C). While this range matches typical battery inlet temperatures (15–35 °C) [35], the large deviation from Span65’s intrinsic melting point (55 °C) limits latent heat utilization and buffering efficiency.

In the EF matrix, the presence of polar LiTFSI salts reduced non-covalent disturbances, shifting transitions closer to Span65’s intrinsic state (melting 30–50 °C; solidification > 40 °C). However, the resulting high viscosity poses risks of flow resistance and blockage in low-temperature regions.

In the EF6 matrix, this optimized system demonstrated ideal characteristics: melting concentrated at 30–40 °C and a broad solidification gradient (32 °C down to 10 °C). This profile matches electronic heat dissipation ranges while facilitating continuous thermal load management. Balancing thermal performance, rheology, and compatibility, EF6 was selected as the base fluid (Table 1).

**Figure 5:**
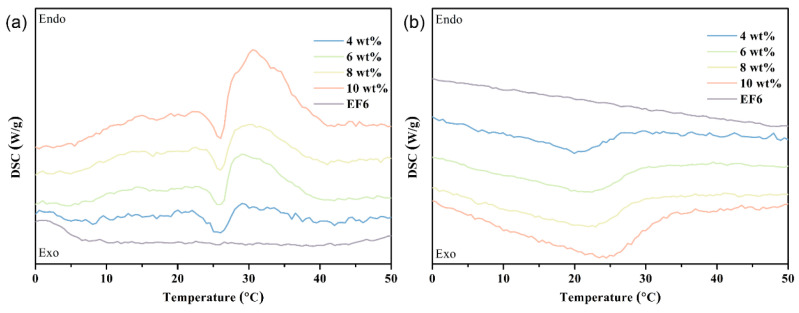
DSC thermograms of PCE with varying Span65 concentrations: (**a**) DSC endothermic curves; (**b**) DSC exothermic curves.

During heating, the coolant exhibits a primary endothermic peak between 25 °C and 40 °C, significantly lower than that of pure Span65 (55 °C), with a broadened, asymmetric profile (Figure 5). This shift is attributed to strong intermolecular interactions between the EF matrix and Span65 molecules (e.g., van der Waals forces, dipole-induced dipoles, and localized hydrogen bonding). These interactions weaken Span65’s lattice stability, creating an “interface softening” effect that reduces melting activation energy and triggers premature crystal dissociation. Furthermore, EF molecules infiltrating crystal defects accelerate the melting process. Notably, a shoulder peak near 20 °C indicates the presence of nanoscale Span65 domains. Consistent with the Gibbs–Thomson effect, these smaller particles exhibit depressed melting points due to higher surface energy. This multi-scale crystal distribution, resulting from high-shear emulsification, induces a stepwise phase transition: finer particles melt first, followed by larger domains at the main peak (~32 °C). This gradient behavior across a wide temperature range enables continuous heat absorption, significantly enhancing thermal buffering and mitigating local temperature spikes.

During cooling, the primary exothermic peak shifts to 3–15 °C, well below the solidification point of pure Span65 (45 °C). This significant supercooling arises from two mechanisms: (1) intermolecular forces between EF and Span65 hinder crystal nucleation and recombination, and (2) restricted Brownian motion in the dispersed state slows crystal growth, extending the phase transition down to near 0 °C. While this delay lowers the operating temperature, it offers a critical engineering advantage: it prevents the formation of large, rigid crystal networks, maintaining small crystal structures that avoid excessive flow resistance or blockage at low temperatures. DSC results confirm distinct phase transition peaks, verifying the successful integration of latent heat storage. This reversible heat absorption/release behavior across a broad temperature range, combined with convective heat transfer, provides robust thermal stability and effectively prevents thermal runaway (Figure 5).

Although latent heat quantification is not the primary focus, DSC results confirm the presence of a distinct phase transition peak, verifying successful integration of thermal energy storage functionality.

### 3.3. Microstructural Characterization of the Phase-Change Component in PCE

**Figure 6:**
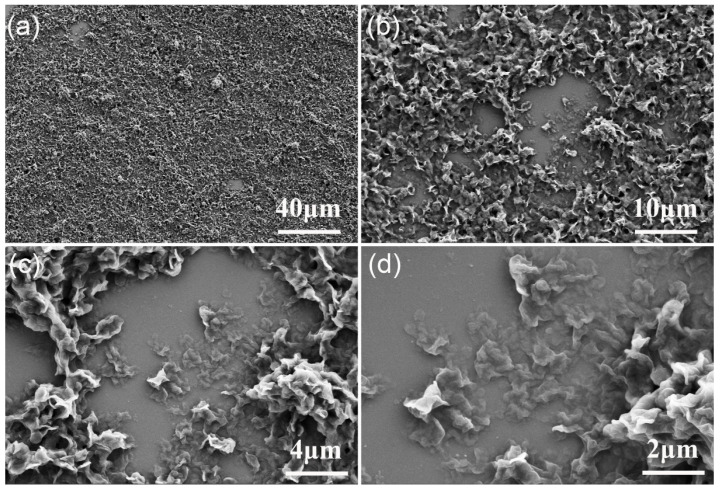
The SEM images of Span65-4wt% at (**a**) 500×, (**b**) 2000×, (**c**) 5000×, and (**d**) 10,000×.

Scanning Electron Microscopy (SEM) reveals that Span65 within the EF6-based phase change coolant forms a distinctive three-dimensional network composed of interlinked petal-like lamellae (0.5–2 μm) interspersed with abundant porous voids (Figure 6). These pores serve a dual function: accommodating the coolant matrix and physically anchoring Span65 components, thereby suppressing phase separation and enhancing structural stability. Furthermore, each lamella exhibits a secondary hierarchical structure of finer stacked layers, significantly increasing the specific surface area to improve dispersion stability and interfacial compatibility within the non-polar synthetic ester matrix.

This multiscale morphology directly correlates with the complex thermal behaviors observed in DSC analysis, such as broadened phase change intervals and the coexistence of primary and shoulder peaks. These features indicate that Span65 does not undergo a single homogeneous transition; instead, it experiences a multistage phase change mechanism driven by the sequential melting or crystallization of structural units at different scales. This established microstructure–thermal behavior relationship provides critical morphological evidence for understanding the coolant’s thermal response and lays a foundational basis for optimizing high-efficiency thermal management systems.

**Figure 7:**
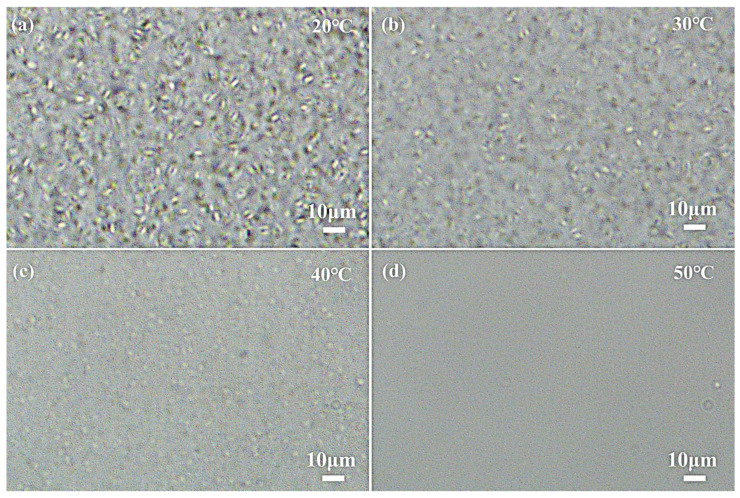
Optical microscopy images of the PCE (Span65, 4 wt%) at various temperatures: (**a**) 20 °C (**b**) 30 °C (**c**) 40 °C (**d**) 50 °C.

In situ optical microscopy observations of the emulsion at different temperatures are presented in Figure 7. At 20 °C (Figure 7a), the presence of abundant disc-shaped PCM particles was noted, corroborating the morphological features observed in SEM images. Given the diffraction limit of optical microscopy, detection was restricted to particles in the micron range. When the temperature was raised to 30 °C (Figure 7b), a marked reduction in the population of observable particles was evident, signifying extensive size reduction and the onset of melting. With further heating (Figure 7c,d), the particles underwent rapid melting and vanished completely, confirming the full liquefaction of the PCM and its homogeneous dispersion in the continuous oil phase.

**Figure 8:**
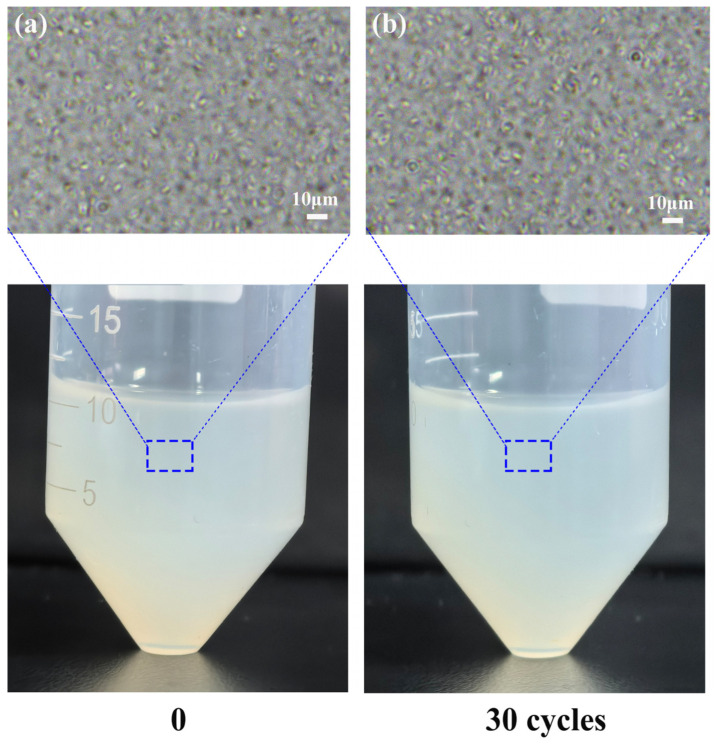
Macroscopic appearance and microstructural morphology of the PCE (Span 65, 4 wt%) before (**a**) and after (**b**) following 30 thermal cycling tests.

To simulate long-term charging and discharging processes, the thermal cycling stability of the PCE (Span65-4wt%) was investigated over 30 cycles within a temperature range of 20 °C to 50 °C. A dwell time of 10 min was maintained at both the maximum and minimum temperature extremes to ensure the completion of the phase transition process. No visible degradation or phase separation was observed in the emulsion after 30 thermal cycles in Figure 8; both its macroscopic appearance and optical microstructure remained intact. This confirms the robust thermal reliability of the prepared PCM.

### 3.4. Rheological Behavior Analysis of PCE

Fluid viscosity is a critical determinant of pumping energy, mass transfer, and heat transport efficiency. This study systematically characterizes the rheological behavior of EF6-based coolants containing 4–10 wt% Span65 across temperatures of 0–50 °C and shear rates of 0.01–1000 s^−1^. Results indicate that viscosity increases monotonically with Span65 concentration at all temperatures, with all samples exhibiting typical shear-thinning (non-Newtonian) behavior.

**Figure 9:**
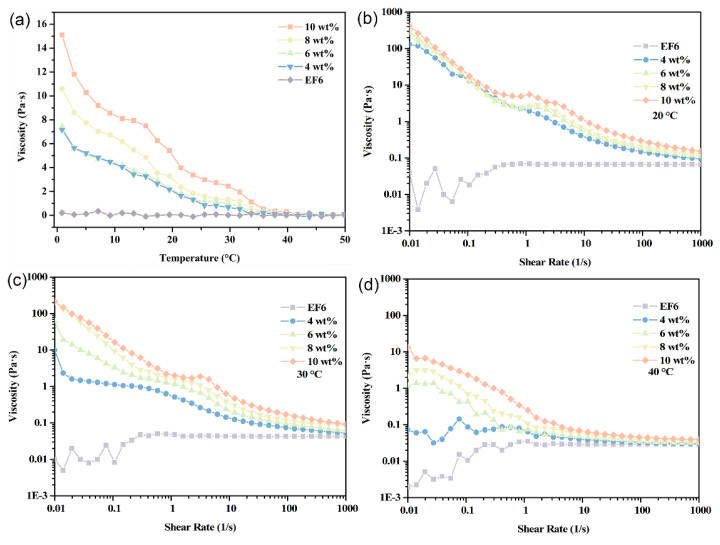
Viscosity–temperature dependence (**a**) and shear-rate-dependent viscosity at selected temperatures—(**b**) 20 °C, (**c**) 30 °C, and (**d**) 40 °C—for phase-change coolants with varying Span65 contents.

Within the lower temperature range (0–20 °C), Span65 exists as solid microcrystals, and system viscosity is governed primarily by the base oil (EF6). While EF6’s low viscosity ensures a gradual decline with rising temperature, all Span65-containing samples exhibit higher viscosity than pure EF6. Notably, samples with ≥6 wt% Span65 display a distinct viscosity plateau near 1 s^−1^, indicating localized Newtonian flow. This phenomenon arises from the dynamic stabilization of a particle network formed via orientation rearrangement under low shear. In contrast, the 4 wt% sample lacks this plateau due to insufficient particle density to form an effective structural network (Figure 9).

**Figure 10:**
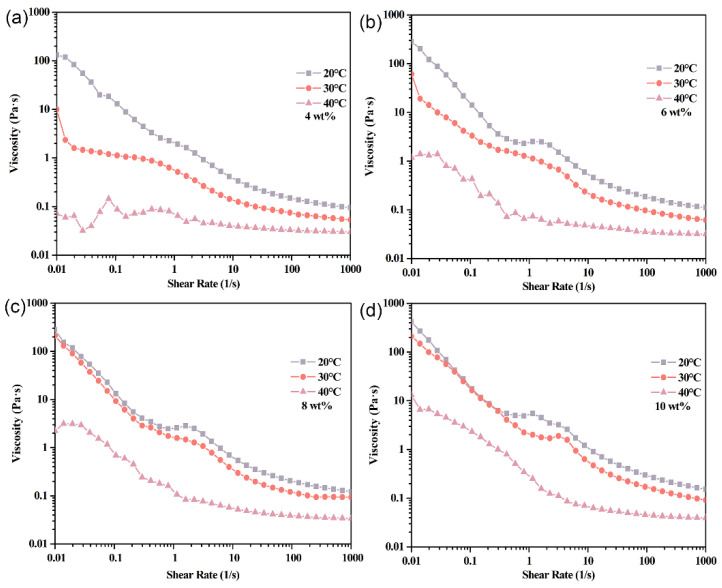
Viscosity as a function of shear rate for phase-change coolants with varying Span65 loadings—(**a**) 4 wt%, (**b**) 6 wt%, (**c**) 8 wt%, and (**d**) 10 wt%—measured at three distinct temperatures: 20 °C, 30 °C, and 40 °C.

Upon reaching approximately 30 °C, as Span65 begins to melt (confirmed by DSC), system viscosity declines rapidly. The reduction in solid-phase resistance lowers overall viscosity across all concentrations. Interestingly, the 4 wt% sample re-emerges with a transient low-shear plateau, likely because reduced viscosity facilitates easier particle rearrangement, temporarily stabilizing the rheological structure despite weaker interparticle forces (Figure 10).

Further elevation to 40 °C shows, according to DSC curves, complete phase transition of all Span65 components, transforming the system into a liquid–liquid dispersion state. At this point, viscosity markedly decreases: at low shear rates, the 4 wt% sample’s viscosity reaches levels comparable to pure EF6 at 20 °C; viscosities of 6 wt% and 8 wt% samples decline by approximately two orders of magnitude compared to those at 20 °C; even under high loading conditions (10 wt%), viscosity reduces by one order of magnitude, underscoring the residual influence of fully melted Span65 on system rheology. In the high shear rate regime (>100 s^−1^), all Span65-containing samples’ rheological behaviors converge towards that of the EF6 matrix, displaying highly similar shear response characteristics (Figure 10). This suggests that under intense shear, the additional contribution of molten Span65 to flow resistance becomes negligible, allowing the system to maintain equivalent pumping performance and heat-mass transfer efficiency as the base coolant. Thus, the viscosity convergence reflects the transition from a structured solid-in-oil dispersion to a stable liquid-in-oil emulsion, not structural collapse. The system retains its emulsion character throughout the thermal cycle, enabling reliable long-term operation.

### 3.5. Rheological Behavior Mechanism of PCE

**Figure 11:**
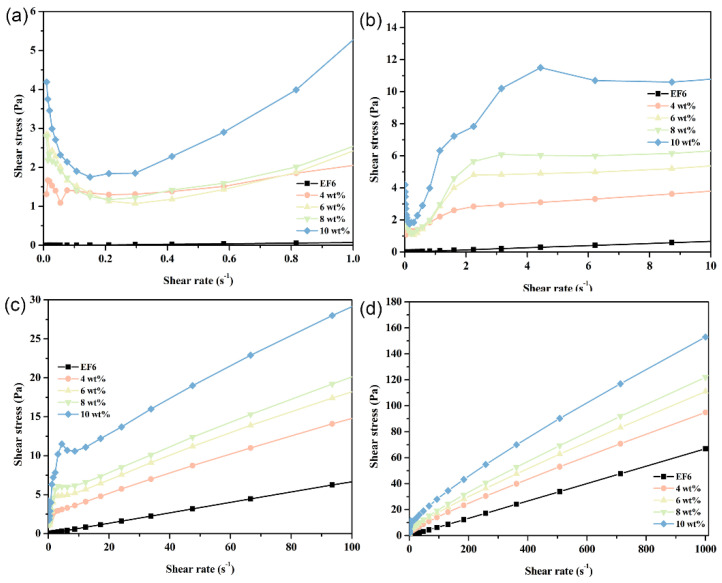
Shear stress as a function of shear rate for phase-change coolants with varying Span65 contents at 20 °C, illustrating: (**a**) Stage I; (**b**) Stage II; (**c**) Stage III; and (**d**) the overall trend.

At 20 °C, the rheological behavior of phase change coolants exhibits pronounced stage-wise transitions across varying shear rates. Specifically, within the shear rate range of 0 to 1.0 s^−1^ (designated as Stage I), the system demonstrates typical pseudoplastic behavior—characterized by shear-thinning. As the shear rate increases to the interval of 1.0–10.0 s^−1^ (Stage II), the material transitions to dilatant (shear-thickening) fluid characteristics. Beyond a shear rate of 10.0 s^−1^ (Stage III), the system adopts Bingham plastic behavior, marked by a finite yield stress followed by near-Newtonian flow. This triphasic rheological response is particularly prominent in formulations with high Span65 loadings; as the Span65 concentration decreases, the pseudoplastic and dilatant features progressively diminish, giving way to dominant Bingham plastic characteristics. In stark contrast, the EF6 matrix displays quasi-Newtonian behavior throughout the entire tested shear rate range, a trait that remains invariant across different temperatures. This observation strongly suggests that the emergence of the aforementioned multistage rheological profile is primarily attributable to the presence of Span65 and its structural influence on the composite system (Figure 11). Rheological tests were repeated in triplicate for the 10 wt% PCE at 20 °C, where the most significant non-Newtonian behavior was observed; other conditions showed stable and reproducible responses, and representative curves are presented (Figures S2 and S3).

**Figure 12:**
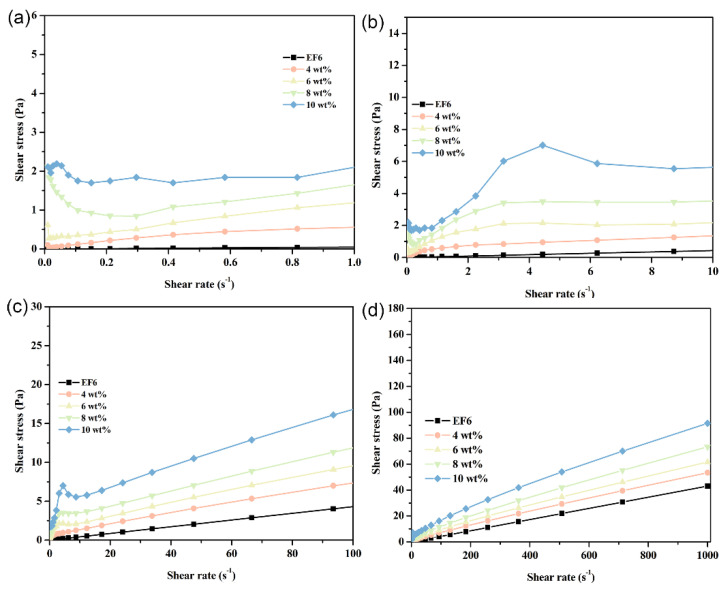
Shear stress as a function of shear rate for phase-change coolants with varying Span65 contents at 30 °C, depicting: (**a**) Stage I; (**b**) Stage II; (**c**) Stage III; and (**d**) the overall trend.

With increasing temperature, the Span65 component within the coolant progressively approaches its melting point, inducing substantial alterations in rheological properties. Notably, the overall shear stress declines, a consequence of partial Span65 melting that reduces internal resistance to flow. At 30 °C, samples with high Span65 content (8 wt% and 10 wt%) exhibit markedly attenuated Stage I and Stage II signatures, whereas those with lower Span65 loadings (4 wt% and 6 wt%) predominantly conform to the rheological pattern of Stage III. This trend is corroborated by DSC analysis: at 30 °C, the majority of Span65 in the low-concentration samples has already undergone phase transition into the liquid state, while in the high-concentration counterparts, melting has only just commenced, leaving a significant fraction of Span65 in the solid phase and thereby preserving certain low-temperature rheological traits. This divergence underscores the critical role of Span65 concentration in governing the rheological response of phase change coolants, as well as the pivotal regulatory effect of temperature on both phase transition kinetics and hydrodynamic performance (Figure 12).

**Figure 13:**
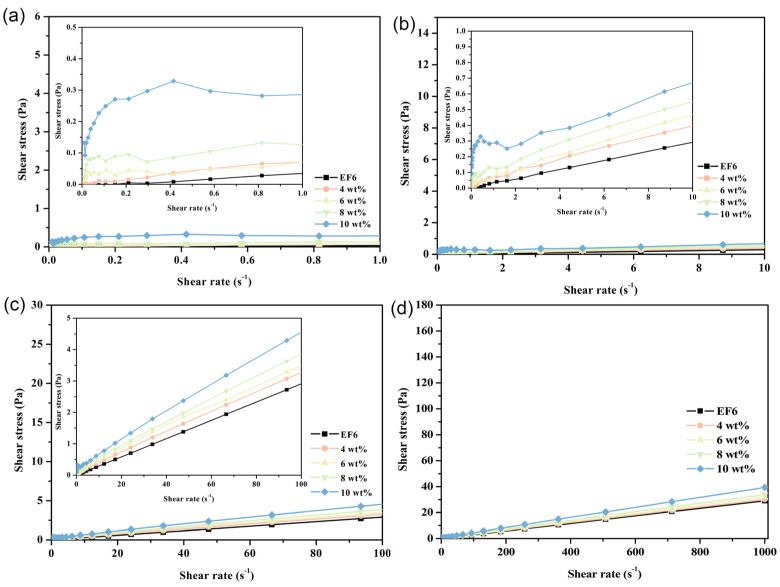
Shear stress as a function of shear rate for phase-change coolants with varying Span65 contents at 40 °C, depicting: (**a**) Stage I; (**b**) Stage II; (**c**) Stage III; and (**d**) the overall trend.

Upon reaching a temperature of 40 °C, as analyzed by DSC curves, the phase transition process of the sample is largely completed, with shear stress significantly decreasing across all shear rates. Except for high concentration (10 wt%) Span65 components, other samples exhibit typical Bingham plastic fluid characteristics and gradually align with the rheological behavior curve of the EF6 matrix. Notably, samples containing 4 wt% Span65 display rheological properties strikingly similar to EF6, consistent with variations in endothermic peak changes in DSC curves, indicating that at this concentration, the melting behavior of Span65 minimally impacts the overall system’s rheological properties (Figure 13).

The observed rheological and thermal behaviors originate from a heterogeneous microenvironment governed by multilevel intermolecular forces, where higher Span65 loadings enhance interactions to form larger, more stable crystals that nonetheless exhibit significantly depressed melting points (~32 °C) compared to pure Span65 (55 °C) or Span65 in unmodified EF (~40 °C). This phenomenon is driven by “matrix confinement,” wherein the polar, hydrogen-bond-rich synthetic ester matrix forms ordered local structures (e.g., micelles or networks) with Span65, creating differentiated thermodynamic microdomains that subject the phase change material to “confined melting,” thereby inducing gradient phase transitions and the characteristic staged rheological responses.

This study introduces EF6, a novel PCE derived from modified ESO and synthetic esters, which establishes itself as a premier candidate for next-generation thermal management of high-power-density electronics by synergizing intrinsic safety, scalable fabrication, intelligent rheology, and superior performance. Unlike water-based fluids [36], EF6 eliminates corrosion and short-circuit risks to enable safe direct immersion cooling, while its liquid–liquid interfacial self-assembly strategy (“high-energy emulsification-ultrasonic temperature-controlled crystallization”) bypasses the complexity and fragility of traditional micro-encapsulation [37] to ensure biodegradability and industrial scalability. Furthermore, the system exhibits intelligent rheological duality—maintaining high viscosity at rest for exceptional storage stability while demonstrating pronounced shear-thinning under flow to minimize pumping energy—thereby overcoming the traditional trade-offs between thermal performance, fluidity, and environmental compatibility through broad-temperature adaptive heat absorption and low-energy transport.

## 4. Conclusions

This study presents a multifunctional phase-change coolant (EF6 + Span65), integrating a modified oil-soluble thermally conductive enhancer (EF6) with Span65. The system achieves synergistic enhancement of thermal management performance while preserving high electrical insulation (σ < 10^−9^ S cm^−1^) and thermal stability. Key features include: (i) linearly enhanced thermal conductivity with increasing EF6 content; (ii) precise tuning of the phase change window to 25~40 °C—ideal for immersion cooling of Li-ion batteries and high-power electronics; (iii) gradient, multi-stage latent heat absorption/release over a broad temperature range, boosting thermal buffering and transient load handling; and (iv) excellent fluidity at operational temperatures for reliable pumpability. Mechanistically, polar functional groups in EF6 modulate Span65 crystallinity via interfacial interactions, lowering melting points, while multiscale Span65 dispersion yields heterogeneous microstructures and multi-peak DSC profiles, indicative of hierarchical phase transitions. Rheologically, the system exhibits strong temperature- and phase-dependent behavior: non-Newtonian at low temperatures (solid Span65) with shear-thinning fluidity, and near-Newtonian at high temperatures (molten Span65), facilitating convective heat transfer and energy-efficient circulation. This work establishes a rational design strategy for constant-temperature thermal management materials with tailored thermophysical properties. However, it is important to acknowledge that the current study is primarily limited to laboratory-scale characterization. The long-term stability under dynamic operating conditions and the scalability of the emulsification process require further validation. Future research will, therefore, focus on extending the cycling tests to thousands of iterations, optimizing the formulation for industrial-scale production, and validating the thermal performance in actual immersion cooling prototypes. These steps are crucial for translating this promising laboratory innovation into a viable commercial solution for next-generation thermal management.

## Figures and Tables

**Table 1:** Parameters of the DSC peak profiles for the Span65 and its formulations in different base fluids—SE, EF, and EF6—following heating and cooling cycles.

	Melting				Solidification				
Sample	Onset Temp/°C	Peak Temp/°C	End Set Temp/°C	Mushy Zone/°C	Onset Temp/°C	Peak Temp/°C	End Set Temp/°C	Mushy Zone/°C	Subcooling/°C
span	44.4	55.0	59.4	15.0	56.4	45.5	33.3	13.1	9.5
SE	0	17.1	25.5	25.5	26.7	16.1	——	——	1.0
EF	27.5	39.0	48.5	21.0	37.6	33.0	——	——	6.0
EF6	26.1	30.5	40.1	14.0	33.1	23.0	——	——	7.5

## Data Availability

The original contributions presented in the study are included in the article/Supplementary Material, further inquiries can be directed to the corresponding author.

## Supplementary Materials

The following supporting information can be downloaded at: https://www.mdpi.com/article/10.3390/ma19071330/s1.
