# Supplementary material for: Oil-Based Phase Change Emulsions Endowed with High Thermal Conductivity and Responsive Rheological Behavior
Source: Materials (Basel). 2026 Mar 27;19(7):1330. doi: 10.3390/ma19071330 (PMC13073820; doi:10.3390/ma19071330)
Supplement: Supplementary file 1 [file materials-19-01330-s001.zip › materials-4171827-supplementary.pdf]

Oil-based phase change emulsions endowed with high thermal conductivity and responsive rheological behavior

Yihua Qian, Qing Wang, Yaohong Zhao*, Zhi Li

Guangdong Key Laboratory of and Electric Power Equipment Reliability, Electric Power Research Institute of Guangdong Power Grid Co., Ltd., Guangzhou, China

* Corresponding author: zhaoyaohong@gddky.csg.cn

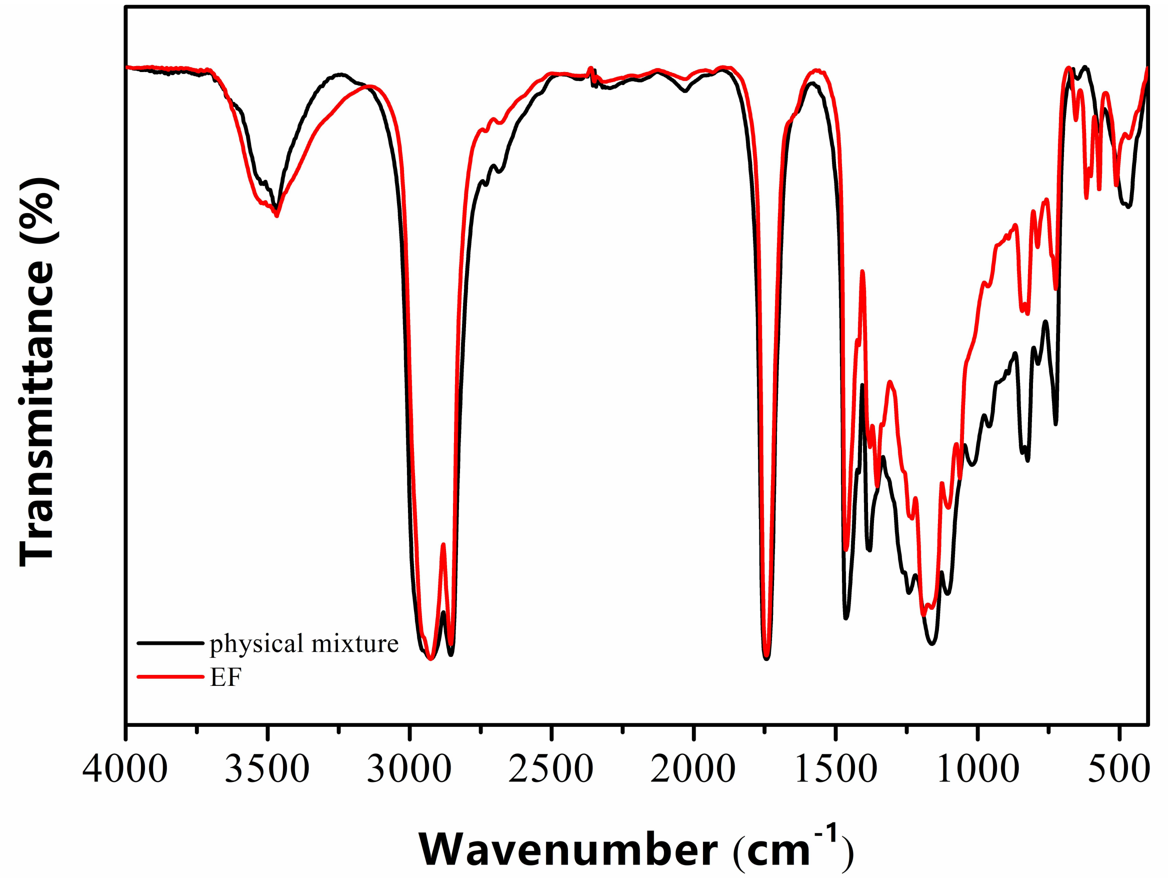

Figure S1 The FTIR spectrum for Physical Mixture of ESO and LiTFSI vs. Reaction-Modified EF

Shear Rate (1/s) 20℃	Shear stress (Pa) EF6	Shear stress (Pa) 4 wt%	Shear stress (Pa) 6 wt%	Shear stress (Pa) 8 wt%	Shear stress (Pa) 10 wt%
0.01	-3.57E-03	1.31E+00	2.84E+00	2.80E+00	4.19E+00
0.014	5.34E-05	1.67E+00	2.83E+00	2.18E+00	3.75E+00
0.0197	7.64E-03	1.64E+00	2.39E+00	2.34E+00	3.46E+00
0.0276	2.37E-03	1.53E+00	2.43E+00	2.16E+00	2.99E+00
0.0387	3.14E-03	1.40E+00	2.28E+00	2.11E+00	2.71E+00
0.0544	3.45E-04	1.09E+00	2.01E+00	1.91E+00	2.32E+00
0.0763	1.98E-03	1.41E+00	1.66E+00	1.73E+00	2.14E+00
0.107	1.95E-03	1.40E+00	1.52E+00	1.41E+00	1.90E+00
0.15	5.15E-03	1.34E+00	1.33E+00	1.25E+00	1.75E+00
0.211	8.00E-03	1.30E+00	1.13E+00	1.17E+00	1.84E+00
0.296	1.64E-02	1.31E+00	1.07E+00	1.22E+00	1.85E+00
0.415	2.68E-02	1.38E+00	1.18E+00	1.42E+00	2.28E+00
0.582	3.86E-02	1.51E+00	1.43E+00	1.59E+00	2.90E+00
0.816	5.70E-02	1.85E+00	1.87E+00	2.01E+00	3.99E+00
1.15	7.98E-02	2.21E+00	2.87E+00	2.97E+00	6.32E+00
1.61	1.07E-01	2.60E+00	4.00E+00	4.59E+00	7.23E+00
2.25	1.49E-01	2.84E+00	4.81E+00	5.65E+00	7.83E+00
3.16	2.14E-01	2.94E+00	4.83E+00	6.09E+00	1.02E+01
4.44	2.99E-01	3.10E+00	4.90E+00	6.03E+00	1.15E+01
6.22	4.17E-01	3.31E+00	4.98E+00	6.00E+00	1.07E+01
8.73	5.86E-01	3.62E+00	5.20E+00	6.15E+00	1.06E+01
12.3	8.21E-01	4.11E+00	5.69E+00	6.59E+00	1.11E+01
17.2	1.15E+00	4.79E+00	6.46E+00	7.36E+00	1.22E+01
24.1	1.61E+00	5.74E+00	7.55E+00	8.51E+00	1.37E+01
33.8	2.26E+00	7.01E+00	9.12E+00	1.01E+01	1.60E+01
47.5	3.18E+00	8.74E+00	1.12E+01	1.24E+01	1.90E+01
66.6	4.46E+00	1.10E+01	1.39E+01	1.53E+01	2.29E+01
93.5	6.25E+00	1.41E+01	1.74E+01	1.92E+01	2.80E+01
131	8.62E+00	1.81E+01	2.20E+01	2.45E+01	3.46E+01
184	1.23E+01	2.34E+01	2.83E+01	3.14E+01	4.33E+01
258	1.72E+01	3.05E+01	3.64E+01	4.06E+01	5.48E+01
362	2.42E+01	4.00E+01	4.76E+01	5.28E+01	7.00E+01
508	3.39E+01	5.31E+01	6.28E+01	6.94E+01	9.03E+01
713	4.77E+01	7.08E+01	8.33E+01	9.20E+01	1.17E+02
1,000	6.69E+01	9.49E+01	1.11E+02	1.22E+02	1.53E+02

Shear Rate (1/s) 30℃	Shear stress (Pa) EF6	Shear stress (Pa) 4 wt%	Shear stress (Pa) 6 wt%	Shear stress (Pa) 8 wt%	Shear stress (Pa) 10 wt%
0.01	4.13E-03	9.99E-02	6.12E-01	2.11E+00	2.11E+00
0.014	5.01E-03	3.30E-02	2.69E-01	1.84E+00	2.09E+00
0.0197	1.71E-03	3.15E-02	2.80E-01	1.78E+00	1.96E+00
0.0276	1.12E-03	4.07E-02	2.77E-01	1.61E+00	2.14E+00
0.0387	1.08E-03	5.36E-02	3.07E-01	1.46E+00	2.19E+00
0.0544	5.43E-04	7.07E-02	3.29E-01	1.34E+00	2.14E+00
0.0763	1.86E-03	9.17E-02	3.21E-01	1.15E+00	1.90E+00
0.107	8.97E-04	1.21E-01	3.56E-01	9.92E-01	1.75E+00
0.15	3.83E-03	1.60E-01	3.69E-01	9.28E-01	1.70E+00
0.211	7.03E-03	2.17E-01	4.39E-01	8.51E-01	1.75E+00
0.296	1.40E-02	2.85E-01	5.02E-01	8.47E-01	1.84E+00
0.415	1.85E-02	3.65E-01	6.72E-01	1.08E+00	1.70E+00
0.582	2.93E-02	4.46E-01	8.41E-01	1.21E+00	1.84E+00
0.816	4.06E-02	5.18E-01	1.06E+00	1.43E+00	1.84E+00
1.15	5.46E-02	5.92E-01	1.29E+00	1.83E+00	2.31E+00
1.61	6.94E-02	6.84E-01	1.57E+00	2.37E+00	2.86E+00
2.25	9.81E-02	7.87E-01	1.77E+00	2.89E+00	3.84E+00
3.16	1.40E-01	8.39E-01	2.12E+00	3.41E+00	6.02E+00
4.44	1.96E-01	9.49E-01	2.15E+00	3.49E+00	7.01E+00
6.22	2.71E-01	1.08E+00	2.03E+00	3.45E+00	5.87E+00
8.73	3.78E-01	1.26E+00	2.08E+00	3.46E+00	5.55E+00
12.3	5.30E-01	1.53E+00	2.35E+00	3.67E+00	5.79E+00
17.2	7.42E-01	1.90E+00	2.81E+00	4.09E+00	6.40E+00
24.1	1.04E+00	2.42E+00	3.45E+00	4.75E+00	7.37E+00
33.8	1.46E+00	3.12E+00	4.34E+00	5.71E+00	8.71E+00
47.5	2.04E+00	4.07E+00	5.51E+00	7.05E+00	1.05E+01
66.6	2.87E+00	5.33E+00	7.06E+00	8.88E+00	1.29E+01
93.5	4.02E+00	7.00E+00	9.08E+00	1.13E+01	1.61E+01
131	5.64E+00	9.00E+00	1.18E+01	1.45E+01	2.02E+01
184	7.89E+00	1.22E+01	1.53E+01	1.88E+01	2.56E+01
258	1.11E+01	1.63E+01	2.00E+01	2.43E+01	3.26E+01
362	1.56E+01	2.18E+01	2.63E+01	3.18E+01	4.18E+01
508	2.19E+01	2.93E+01	3.47E+01	4.18E+01	5.40E+01
713	3.07E+01	3.95E+01	4.61E+01	5.52E+01	7.00E+01
1,000	4.31E+01	5.34E+01	6.16E+01	7.33E+01	9.15E+01

Shear Rate (1/s) 40℃	Shear stress (Pa) EF6	Shear stress (Pa) 4 wt%	Shear stress (Pa) 6 wt%	Shear stress (Pa) 8 wt%	Shear stress (Pa) 10 wt%
0.01	-1.89E-03	7.17E-04	1.19E-02	2.25E-02	1.32E-01
0.014	-1.45E-02	2.24E-03	1.96E-02	4.40E-02	9.25E-02
0.0197	-5.35E-03	1.27E-03	2.61E-02	6.13E-02	1.32E-01
0.0276	-1.99E-03	-2.29E-03	3.85E-02	8.11E-02	1.49E-01
0.0387	-3.42E-03	4.75E-04	3.11E-02	7.97E-02	1.76E-01
0.0544	-2.76E-03	4.25E-03	3.84E-02	8.35E-02	1.95E-01
0.0763	-1.89E-03	1.10E-02	3.22E-02	8.94E-02	2.27E-01
0.107	-1.93E-03	9.34E-03	4.60E-02	7.43E-02	2.49E-01
0.15	6.59E-05	9.31E-03	2.88E-02	8.93E-02	2.71E-01
0.211	4.73E-03	1.53E-02	4.43E-02	9.56E-02	2.72E-01
0.296	3.15E-03	2.23E-02	4.01E-02	7.18E-02	2.97E-01
0.415	8.26E-03	3.65E-02	3.00E-02	8.55E-02	3.29E-01
0.582	1.66E-02	4.97E-02	5.00E-02	1.05E-01	2.97E-01
0.816	2.78E-02	6.57E-02	5.37E-02	1.32E-01	2.82E-01
1.15	4.05E-02	7.39E-02	8.48E-02	1.22E-01	2.89E-01
1.61	4.64E-02	7.79E-02	1.02E-01	1.34E-01	2.51E-01
2.25	6.29E-02	1.25E-01	1.18E-01	1.87E-01	2.82E-01
3.16	9.56E-02	1.44E-01	1.84E-01	2.43E-01	3.53E-01
4.44	1.31E-01	2.06E-01	2.30E-01	3.10E-01	3.84E-01
6.22	1.82E-01	2.69E-01	3.08E-01	3.91E-01	4.69E-01
8.73	2.56E-01	3.53E-01	4.18E-01	5.01E-01	6.15E-01
12.3	3.57E-01	4.77E-01	5.57E-01	6.42E-01	7.75E-01
17.2	5.01E-01	6.46E-01	7.51E-01	8.35E-01	1.02E+00
24.1	7.01E-01	8.80E-01	1.01E+00	1.10E+00	1.34E+00
33.8	9.84E-01	1.20E+00	1.38E+00	1.47E+00	1.79E+00
47.5	1.38E+00	1.64E+00	1.86E+00	1.98E+00	2.37E+00
66.6	1.94E+00	2.24E+00	2.40E+00	2.68E+00	3.18E+00
93.5	2.72E+00	3.07E+00	3.28E+00	3.63E+00	4.29E+00
131	3.81E+00	4.22E+00	4.51E+00	4.95E+00	5.81E+00
184	5.35E+00	5.82E+00	6.18E+00	6.77E+00	7.89E+00
258	7.49E+00	8.04E+00	8.54E+00	9.28E+00	1.08E+01
362	1.05E+01	1.12E+01	1.18E+01	1.28E+01	1.49E+01
508	1.48E+01	1.55E+01	1.64E+01	1.77E+01	2.05E+01
713	2.08E+01	2.17E+01	2.29E+01	2.44E+01	2.84E+01
1,000	2.91E+01	3.02E+01	3.18E+01	3.38E+01	3.94E+01

Figure S2 Raw rheological data showing shear stress as a function of shear ratefor the formulated phase change coolant at different temperatures. Steady-

shear measurements were performed over a shear rate range of 0.1–1000 s^{-1} at 20 °C, 30 °C, and 40 °C (spanning the phase transition region).

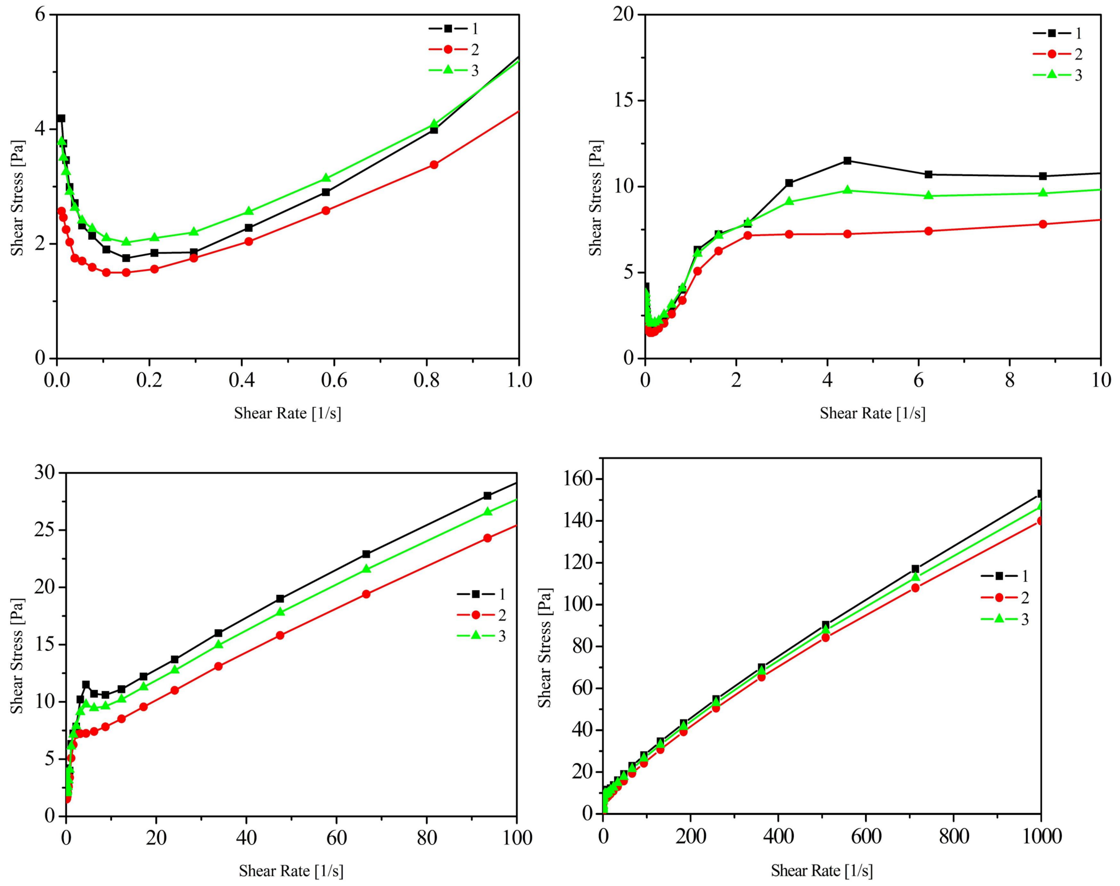

Figure S3: Shear stress as a function of shear rate for phase-change coolants with 10wt% Span65 contents at 20 °C, illustrating: Stage I; Stage II; Stage III; and the overall trend. (The labels 1, 2, and 3 denote the results of three repeated measurements, respectively.)
